# Comparative Genomics of *Aspergillus flavus* S and L Morphotypes Yield Insights into Niche Adaptation

**DOI:** 10.1534/g3.118.200553

**Published:** 2018-10-25

**Authors:** Mana Ohkura, Peter J. Cotty, Marc J. Orbach

**Affiliations:** * School of Plant Sciences, University of Arizona, Tucson, Arizona 85721; † USDA-ARS, School of Plant Sciences, University of Arizona, Tucson, Arizona 85721

**Keywords:** *Aspergillus flavus*, aflatoxin, adaptation, comparative genomics, sclerotia

## Abstract

*Aspergillus flavus*, the primary causal agent for aflatoxin contamination on crops, consists of isolates with two distinct morphologies: isolates of the S morphotype produce numerous small sclerotia and lower numbers of conidia while isolates of the L morphotype produce fewer large sclerotia and abundant conidia. The morphotypes also differ in aflatoxin production with S isolates consistently producing high concentrations of aflatoxin, whereas L isolates range from atoxigenic to highly toxigenic. The production of abundant sclerotia by the S morphotype suggests adaptation for long-term survival in the soil, whereas the production of abundant conidia by the L morphotype suggests adaptation for aerial dispersal to the phyllosphere. To identify genomic changes that support differential niche adaption, the sequences of three S and three L morphotype isolates were compared. Differences in genome structure and gene content were identified between the morphotypes. A >530 kb inversion between the morphotypes affect a secondary metabolite gene cluster and a cutinase gene. The morphotypes also differed in proteins predicted to be involved in carbon/nitrogen metabolism, iron acquisition, antimicrobial defense, and evasion of host immunity. The S morphotype genomes contained more intact secondary metabolite clusters indicating there is higher selection pressure to maintain secondary metabolism in the soil and that it is not limited to aflatoxin production. The L morphotype genomes were enriched in amino acid transporters, suggesting efficient nitrogen transport may be critical in the nutrient limited phyllosphere. These findings indicate the genomes of the two morphotypes differ beyond developmental genes and have diverged as they adapted to their respective niches.

The saprotrophic fungus, *Aspergillus flavus*, is the primary causal agent of contamination in food crops by aflatoxins, highly carcinogenic secondary metabolites produced by members of the genus *Aspergillus* section *Flavi* (Schroeder and Boller 1973; Klich 2002; Klich 2007; Mehl *et al.*, 2012). Aflatoxin contamination causes both economic losses and health problems in both humans and animals. This is a serious problem in developing countries where aflatoxin contamination is not regulated; aflatoxin-contaminated food and feed result in loss of export sales and health problems ranging from stunting of growth, immune suppression, cancer and in acute cases, death (Goldblatt 1969; Bennett and Klich 2003; Probst *et al.*, 2007; Richard *et al.*, 2003; Williams *et al.*, 2004). Repeated aflatoxicosis outbreaks have occurred in Kenya, and in 2004, one of the largest documented outbreaks resulted in a total of 317 cases being reported and 125 deaths (CDC, 2004; Probst *et al.*, 2007). In developed countries, regulations for aflatoxin levels in food protect consumers from aflatoxin poisoning, but great economic losses are incurred as contaminated crops must be destroyed or de-contaminated (Richard *et al.*, 2003; Mitchell *et al.*, 2016).

Although *A. flavus* is notorious for its ability to produce aflatoxin, isolates within the species vary in levels of toxin production and atoxigenic isolates are not uncommon. Within *A. flavus* are isolates with two distinct morphologies, the S (small sclerotia) and L (large sclerotia) morphotypes (Cotty 1989), characterized by sclerotial size. S morphotype isolates produce numerous small sclerotia and conidiate poorly, whereas L morphotype isolates produce relatively fewer large sclerotia and abundant conidia (Cotty 1989; Figure 1). The morphotypes differ in toxigenicity as well: S isolates are consistently toxigenic, whereas L isolates vary greatly in toxin production ranging from atoxigenic to highly toxigenic isolates (Saito *et al.*, 1986; Cotty 1989). On average, S isolates produce higher levels of aflatoxin than L isolates (Cotty 1989; Cotty 1997; Probst *et al.*, 2010) and have etiological implications as causal agents in outbreaks (Cotty 1997; Garber and Cotty 1997; Bock *et al.*, 2004; Probst *et al.*, 2007; Cotty *et al.*, 2008). Atoxigenic L isolates are of particular importance as successful biological control agents that outcompete toxigenic isolates from contaminating crops (Garber and Cotty 1997; Cotty *et al.*, 2008; Mehl *et al.*, 2012).

**Figure 1:**
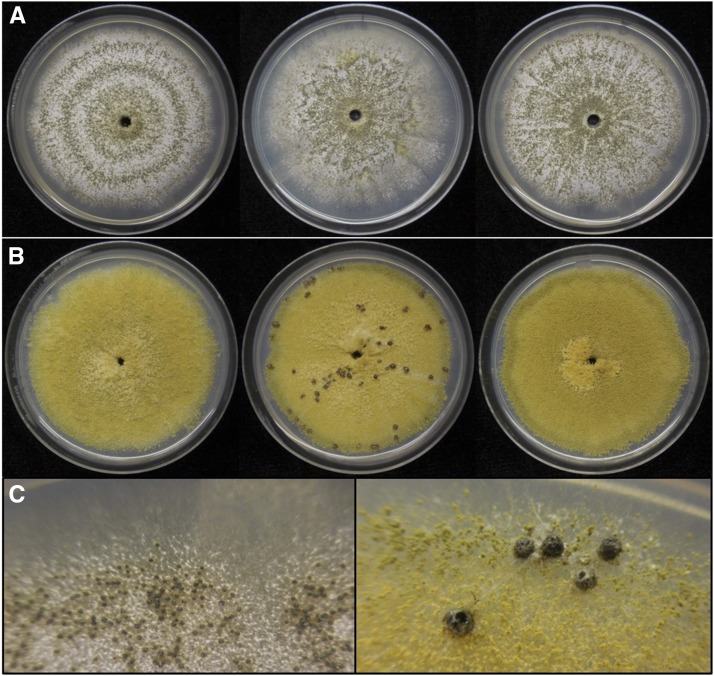
Colony morphology of *A. flavus* isolates used in genomic analyses of this study. Cultures were grown on complete media at 30 °C in the dark for 10 days. (A) Colonies of S morphotype isolates displaying limited conidial sporulation and abundant production of small sclerotia. From left to right; AF12, AF70, AZS. (B) Colonies of L morphotype isolates displaying abundant conidial sporulation and limited or no production of large sclerotia. From left to right; BS01, DV901, MC04. (C) Closer view of small sclerotia produced by AZS (left) and large sclerotia produced by DV901 (right).

Developmental and biochemical differences between *A. flavus* S and L isolates suggest they are adapted to different niches: S isolates produce abundant sclerotia that are advantageous for long-term survival in the soil, whereas L isolates produce fewer sclerotia, but large numbers of conidia that are advantageous for aerial dispersal to the phyllosphere (Mehl *et al.*, 2012). A study by Sweany *et al.* (2011) provides support for this differential reproductive strategy: they report that more than 95% of isolates recovered from maize kernels in a study in Louisiana were of L morphotype, whereas only 44% of soil isolates were of L morphotype with the remainder consisting of S morphotype. A similar pattern was observed when comparing the frequency of S isolates and L isolates recovered from maize kernels and groundnut in Malawi (Ching’anda *et al.*, 2016). Differences in aflatoxin toxigenicity observed between the morphotypes also suggest differential niche adaptation; S isolates are consistently highly toxigenic, while L isolates vary in their toxigenicity including atoxigenic and low toxin-producing isolates. This suggests selection pressure to maintain aflatoxin production varies between the soil and phyllosphere with pressure being high in the soil, leading to the retention of toxigenic S isolates, while it is lower in the phyllosphere allowing the survival of atoxigenic or low toxin-producing L isolates (Mehl *et al.*, 2012). This is also evidenced by lower proportions of atoxigenic isolates recovered from soil than from the phyllosphere (Bilgrami and Choudhary 1993; Sweany *et al.*, 2011).

In this study, we compared the genomes of S and L morphotype isolates to test the hypothesis that there are genomic differences that reflect their adaptation to the soil and phyllosphere respectively. The soil and phyllosphere are contrasting environments in terms of ambient conditions, nutrient availability, and microbial competition. The soil is buffered from changes in the atmosphere in contrast to the phyllosphere where microbes are exposed to high levels of ultraviolet light and fluctuations in temperature (Lindow and Brandl 2003; Whipps *et al.*, 2008; Vorholt 2012; Bulgarelli *et al.*, 2013; Bringel and Couée 2015). Nutrient availability is greater in the soil than the phyllosphere due to the presence of root exudates and degradation of organic matter by microorganisms (Rengel and Marschner 2005; Turner *et al.*, 2013; Dotaniya and Meena 2015). In contrast, the leaf surface is covered by a waxy cuticle layer that limits passive diffusion of water and nutrients (Whipps *et al.*, 2008; Vorholt 2012; Bringel and Couée 2015). In recent years, metagenomic studies of the soil/rhizosphere and phyllosphere microbiomes show evidence that microbial communities that inhabit these niches are different as well (Delmotte *et al.*, 2009; Knief *et al.*, 2012; Lundberg *et al.*, 2012). The soil environment is rich, not only with microbes, but also with small animals such as insects and worms . On the other hand, species diversity on leaf surfaces is relatively low, with bacteria being the most abundant organisms (Lindow and Brandl 2003; Delmotte *et al.*, 2009). Therefore, if the S and L morphotypes are adapted to the soil and phyllosphere respectively, they would require different mechanisms to withstand abiotic stresses, acquire nutrients, and compete or defend against organisms they encounter. Here we identify genomic differences between the *A. flavus* morphotypes that we propose have allowed them to selectively adapt to their respective niches.

## Methods and Materials

### Genome sequencing, assembly, and annotation

The genomes of three S morphotype isolates (AF12, AF70, and AZS; Figure 1; Table S1) and three L morphotype isolates (BS01, DV901, and MC04; Figure 1; Table S1) were 2 × 100 bp paired-end sequenced using the Illumina HiSeq 2000 (San Diego, CA) platform at the Arizona Genomics Institute. Nucleotide coverage for each genome was 40x (AF12), 42x (AF70), 40x (AZS), 120x (BS01), 44x (DV901), and 123x (MC04). The genomes of BS01 and MC04 were resequenced due to low coverage in the first sequencing run. Genomic DNA was extracted from cultures grown in complete medium (1% glucose, 0.3% yeast extract, 0.3% casein hydrolysate) for 3 days at 31°. The extraction was performed using a modified protocol from Kellner *et al.* (2005) by harvesting mycelia by centrifugation and mechanically lysing the cells using a pestle. Sequence reads were quality filtered (phred score > 15 for 4-base sliding window) with Trimmomatic v0.22 (Bolger *et al.*, 2014). Optimal k-mer values to use for assembly were determined using VelvetOptimizer and by assessing the completeness of the assembly against the *A. oryzae* RIB40 genome (a domesticated species of *A. flavus*) using QUAST (Gurevich *et al.*, 2013). Genomes were assembled with Velvet v1.2.10 using following k-mers: 55 (AF12), 51 (AF70), 53 (AZS), 61 (BS01), 57 (DV901), and 63 (MC04).

The genomes were annotated by MAKER v2.31 (Holt and Yandell 2011) using following parameters: A training set of genes was obtained by SNAP (Korf 2004), which was trained using the *A. oryzae* model available at https://github.com/hyphaltip/fungi-gene-prediction-params/blob/master/params/SNAP/aspergillus_oryzae.length.hmm. AUGUSTUS (Holt and Yandell 2011) predicted many gene fusions and thus was not used to predict genes for training. For protein evidence, proteins from *A. oryzae* RIB40 (Aspgd version s01-m08-r20; 11902 proteins) and *A. flavus* NRRL3357 (Uniprot proteins that were reviewed, have protein or transcript evidence, or have homology to other organisms; 1843 proteins) were used. To avoid fragmented gene calls, genes were only predicted on contigs larger than 1 kb. Single exon genes were allowed and only gene models with protein evidence were accepted. To obtain the final set of gene calls, SNAP (Korf 2004) and AUGUSTUS (Holt and Yandell 2011) were trained on the training set of gene models, and MAKER (Holt and Yandell 2011) was run using the same parameters as the training round, but allowing prediction of genes without protein evidence. Gene calls were visualized using Apollo (Lee *et al.*, 2013) to confirm they aligned with the protein evidence. InterProScan 5.14.53 (Jones *et al.*, 2014) and SwissProt 2014_08 release (Boeckmann *et al.*, 2003) were used to predict functions of the gene models.

### Identification of structural variations between morphotypes

To identify large structural variations between the morphotypes, contigs of each genome were assembled into pseudogenomes based on the chromosomes of *A. oryzae* RIB40 using CoGE Synmap (Lyons and Freeling 2008). Synteny analysis was performed by comparing each pseudogenome against the remaining five genomes using Symap v4.2 (Soderlund *et al.*, 2011). In addition, the inversion on chromosome 8 was confirmed by PCR using primers listed in Table 1; amplification of the 5′ half of *tsr1* was used as a positive control.

**Table 1:** Primers used for phylogenetic analysis and confirmation of the inversion on Chromosome 8

Locus	Forward primer	Reverse primer	Product	T_a_ (°C)
5′ half of *tsr1*	OAM1523: GGGTCCAGCGGTGCCAATTCGG	OAM1524: CTCTTCCGGTGCCACAACAGC	≈1148 bp	58
3′ half of *tsr1*	OAM1525: CCGGATCAGACCTCAGCGATGG	OAM1526: CCCTTTGAAACCGCCCATATCAA	≈1210 bp	58
*cmd*	OAM1571: CAGTCTTTGTATCTTTGTTCCTCTCC	OAM1572: CCTGAATGGGGTGTATGATAAACG	≈1319 bp	60
Inversion breakpoint A in S morphotype genomes	OAM1537: GGAACAAGATGCGAGATCCTGG	OAM1533: GAGGAAAATGAATCTAGCCCTGC	≈864 bp	55
Inversion breakpoint B in S morphotype genomes	OAM1538: GCCGCATCTAAGGAGCAGACT	OAM1534: CGGTGTTCTTGCTTGTCCCG	≈1723 bp	58
Inversion breakpoint C in L morphotype genomes	OAM1538: GCCGCATCTAAGGAGCAGACT	OAM1537: GGAACAAGATGCGAGATCCTGG	≈1987 bp	58
Inversion breakpoint D in L morphotype genomes	OAM1533: GAGGAAAATGAATCTAGCCCTGC	OAM1534: CGGTGTTCTTGCTTGTCCCG	≈598 bp	55

To identify smaller structural variations between the S and L morphotypes, paired-end and split read analyses were performed (Rausch *et al.*, 2012). Contigs of AZS and DV901 were used as references for the S and L morphotypes respectively, because they had the highest N50 values indicating the best assemblies. Trimmed reads of AF12, AF70, BS01, DV901, and MC04 were mapped to contigs of AZS, and trimmed reads of AF12, AF70, AZS, BS01, and MC04 were mapped to contigs of DV901 with Bowtie2 v2.2.4 (Langmead and Salzberg 2012). DELLY v0.6.1 (Rausch *et al.*, 2012) was used to identify deletions, duplications, transpositions, and inversions from the mapping output. Structural variants with at least one pair of mapping reads were kept, because structural variations specific to each morphotype were determined by its presence in all three genomes of the morphotype. To identify structural variants that are only present in the genomes of the S morphotype, first, the multiIntersect feature in BEDtools v2.23 (Quinlan and Hall 2010) was used to identify structural variants that AF12, AF70, and AZS had in common against DV901. Next, out of the structural variants that may be unique to the S isolates *vs.* DV901, those that were present in comparisons between DV901 and other L isolates (BS01 and MC04) were eliminated. Only S isolate variants that were not present in the L to L isolate comparisons were defined as S-specific. Similarly, to identify structural variants that are only present in the genomes of the L morphotype, first, the structural variants that BS01, DV901, and MC04 had in common against AZS were identified, and next, out of the variants, those that were present in comparisons of AF12 and AF70 against AZS were removed; thus, only L isolate variants that were not present in the S to S isolate comparisons were defined as L-specific. Deletions larger than 5 kb and all remaining structural variations identified by DELLY were confirmed manually using CoGe GEVO (Lyons and Freeling 2008) as well.

### GO term enrichment and gene family expansion/contraction analyses

GO term enrichment analysis between genomes of the two morphotypes was carried out by the hypergeometric test (p-value < 0.05) in OrthoVenn (Wang *et al.*, 2015). Protein families were determined by Pfam annotations (Finn *et al.*, 2016) from InterProScan (Jones *et al.*, 2014). Protein families that were unique to each morphotype were confirmed by both BLASTn analysis (Altschul *et al.*, 1990) of the genes against the genomes of the other morphotype and via the CoGE genome browser (Lyons and Freeling 2008): if the nucleotide sequence for the protein was present in genomes of the other morphotype, this indicated a possible misannotation in that morphotype, thus the protein family was removed from the list of unique proteins. Functional enrichment of Pfam categories between the two morphotypes was carried out using the hypergeometric test (p-value < 0.05) in GeneMerge v1.4 (Castillo-Davis and Hartl 2003).

### Identification of morphotype-unique proteins

Morphotype-unique proteins were defined as those encoded by a gene in the same position in all three genomes of one morphotype that lacked orthologs in all three genomes of the other morphotype. Proteins from the six genomes were clustered using Orthofinder (Emms and Kelly 2015) and orthologous genes that are positionally conserved were clustered using progressiveMauve (Darling *et al.*, 2010). Clusters defined by both clustering methods were extracted and filtered to those that contain proteins from only the three S morphotype genomes or only the three L morphotype genomes. These clustering approaches can fail to include orthologs that are truncated or misannotated from its appropriate cluster; therefore, the clusters were further filtered by BLASTn analysis (e-value <1e-10; Altschul *et al.*, 1990) against all contigs of the other morphotype. Clusters that contained hits with ≥80% query coverage per subject and ≥80% identity were removed and remaining clusters were designated as morphotype-unique proteins. The chromosomal locations of these morphotype-unique proteins were visualized on their pseudogenomes using CViT v1.2.1 (Cannon and Cannon 2011).

### Identification of genes under selection between morphotypes

To identify genes under selection, the POTION 1.1.2 pipeline (Hongo *et al.*, 2015) was implemented on clusters from Orthofinder (Emms and Kelly 2015) that contained single-copy orthologs from all six genomes with >80% sequence identity. Genes with evidence of recombination were identified with PhiPack (Bruen *et al.*, 2006) and removed using default parameters. Multiple sequence alignment was performed using MUSCLE (Edgar 2004), protein-guided codon alignments were created using a subroutine within POTION (Hongo *et al.*, 2015), sequences were trimmed with trimAL (Capella-Gutiérrez *et al.*, 2009), and phylogenetic tree reconstruction was performed with PhyML (Guindon *et al.*, 2009). Likelihood ratio tests for selection were performed using the m12 (codeml M1a/M2) and m78 (codeml M7/M8) nested site models with PAML codeml (Yang 2007) to compare neutral models with models that allow positive selection. Genes under selection in each genome were identified relative to the three genomes of the other morphotype using a q-value < 0.05 cutoff (corrected p-value for multiple testing) in either the m12 or m78 model test. Genes under selection that are morphotype-specific were identified as genes selected in all three isolates of a morphotype within a cluster.

### Additional annotation of proteins: Secondary metabolite clusters, antibiotic resistance genes, CAZy, and peptidases

Identification of secondary metabolite clusters was performed by SMURF (Khaldi *et al.*, 2010). Proteins that are predicted to confer antibiotic resistance were identified using BLASTp provided in the comprehensive antibiotic resistance database (CARD; Jia *et al.*, 2017) using ‘strict’ as the cutoff. Carbohydrate-active enzymes (CAZy) in each genome were identified by analyzing protein sequences with HMMER3 against the dbCAN database (Yin *et al.*, 2012). Identification of peptidases and peptidase inhibitors was carried out by the MEROPS 10.0 batch BLAST program (Rawlings *et al.*, 2012). Proteins with morphotype-unique CAZy and MEROPS categories were confirmed by BLASTp analysis (Altschul *et al.*, 1990) against the NCBI nr database (NCBI Resource Coordinators 2017) as well: if proteins with similar sequences in the database were annotated with a function different from that suggested by the CAZy or MEROPS categories and the NCBI annotation was functionally validated, the NCBI annotations took precedence.

### Phylogenetic analyses

To evaluate whether the >530 kb inversion may have led to the divergence of S and L morphotypes, a phylogenetic analysis that includes isolates from diverse geographic regions was performed using DNA sequences from the 5′ half of *tsr1* (ribosome biogenesis protein), 3′ half of *tsr1*, and *cmd* (calmodulin) genes (Table 1). Genomic DNA of eight *A. flavus* S isolates, 22 *A. flavus* L isolates, and four outgroup isolates (Table S1; Cotty 1989; Probst *et al.*, 2012; Probst *et al.*, 2014) was extracted from 7 day-old cultures grown at 31° in the dark on V8 agar (5% V8 vegetable juice, 2% NaCl, and 2% agar, pH 5.2). DNA extraction was performed using the method of Callicott and Cotty (2015). Polymerase chain reactions (PCR) of each locus were performed using the primers and annealing temperatures listed in Table 1. The reactions were performed in 25 μL volumes using 3 ng of genomic DNA and Promega GoTaq Green Master Mix (Madison, WI), following the manufacturer’s protocol. Amplicons were sequenced at the University of Arizona Genetics Core using an Applied Biosystems 3730 DNA analyzer. Forward and reverse sequences were edited and assembled using SeqTrace 0.9.0 (Stucky 2012). For each gene, sequences were aligned in MUSCLE (Edgar 2004) and manually edited, trimmed, and assigned codon positions in Mesquite (Maddison and Maddison 2017). The three loci were concatenated and submitted to PartitionFinder v2.1.1 (Lanfear *et al.*, 2016) to infer the best-fitting model of evolution among those available in RAxML (Stamatakis 2006) based on codon position and locus. The concatenated dataset was partitioned into five subsets, which were then implemented in RaxML for maximum likelihood analysis (Stamatakis 2006) using CIPRES (Miller *et al.*, 2010). Outgroup taxa consisted of *A. minisclerotigenes*_,_ and support was assessed using 1000 maximum likelihood bootstrap replicates.

### Data availability

Genome sequences can be accessed at NCBI under the following accession numbers: NLCN00000000 (AF12), NLCM00000000 (AF70), NLCL00000000 (AZS), NLCK00000000 (BS01), NLCJ00000000 (DV901), and NLCI00000000 (MC04). The genomes are listed under BioProject PRJNA393333. Supplemental material available at Figshare: https://doi.org/10.25387/g3.6721511.

## Results

### Genome assembly and annotation

A summary of the genome assemblies and annotations are listed in Table 2. The genome assemblies of the S isolates ranged in size from 38.1 Mb – 38.3 Mb (N50 values of 0.86 Mb – 1.14 Mb), and those of the L isolates ranged in size from 37 Mb – 37.5 Mb (N50 values of 0.93 Mb – 1.03 Mb). The predicted number of genes ranged from 13,368 – 13,374 for the S morphotype genomes, and 13,256 – 13,292 for L morphotype genomes. On average, the S morphotype genomes were larger by 1 Mb and contained 99 more genes. These assemblies are similar in size and in gene numbers to the previously sequenced genomes of *A. flavus* NRRL3357 (L morphotype; Nierman *et al.*, 2015) and *A. oryzae* RIB40 (Machida *et al.*, 2005) that are both 37 Mb in size with 13,485 predicted genes and 12,074 predicted genes, respectively. Predicted repetitive DNA content was slightly higher in the S morphotype genomes ranging from 1.7–1.86% relative to the L morphotype genomes that ranged from 1.29 – 1.49%. These values are considered to be an underestimate of the actual repetitive DNA content because repetitive regions do not assemble well from short sequence reads.

**Table 2:** Summary of genome assembly and annotation

	S morphotype			L morphotype		
	AF12	AF70	AZS	BS01	DV901	MC04
Total size (Mb)	38.1	38.1	38.3	37	37	37.5
Nucleotide coverage	40x	42x	40x	120x	44x	123x
N50 (Mb)	0.86	0.97	1.14	0.93	1.03	0.95
Total contigs (>500bp)	438	365	305	286	267	385
Longest contig (Mb)	2.04	2.2	2.09	2.7	2.11	1.84
GC (%)	47.4	47.3	47.1	48	48	47.6
Repetitive content (%)	1.71	1.7	1.86	1.34	1.29	1.49
Non-coding sequence (Mb)	19.5	19.4	19.7	18.5	18.4	18.9
Total predicted genes	13374	13371	13368	13256	13268	13292

### Structural variations between morphotypes

**Figure 2:**
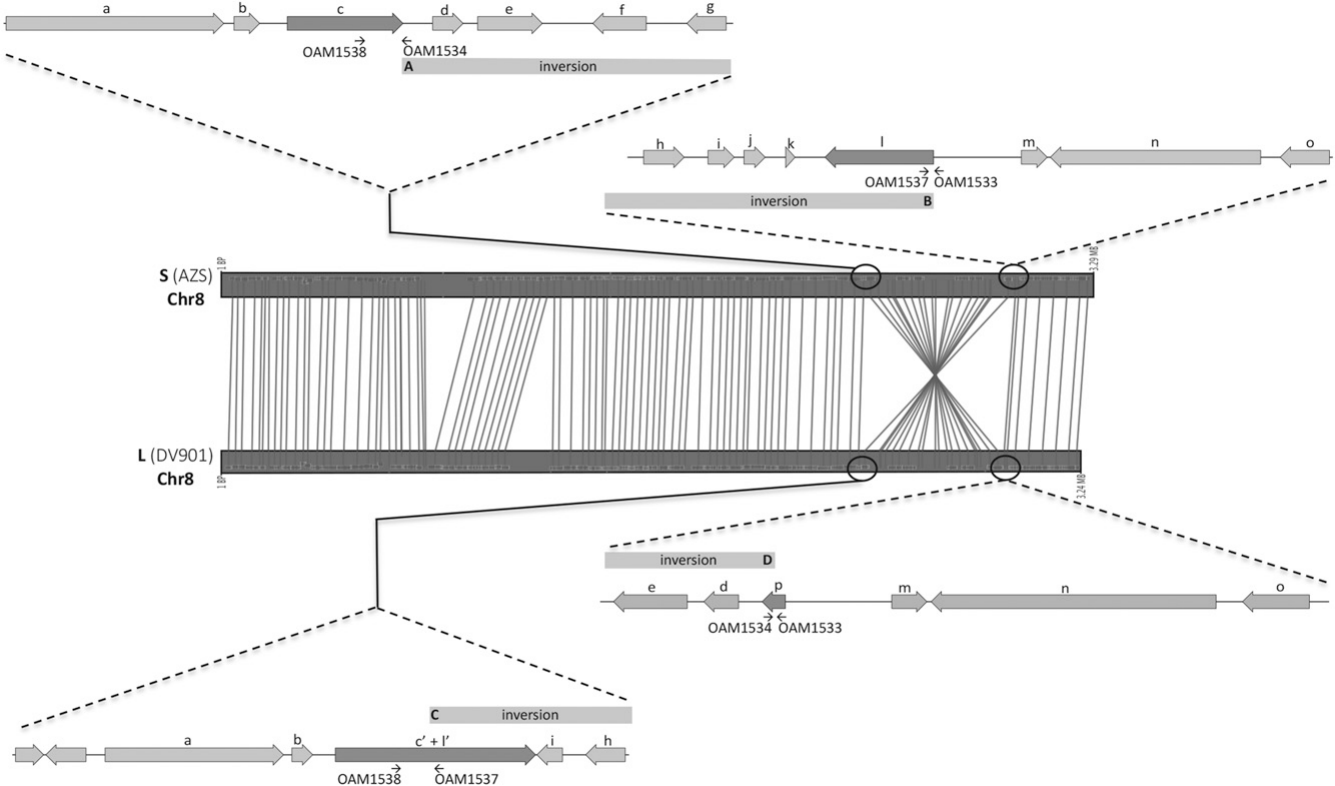
A schematic diagram of the chromosomal inversion on chromosome 8 using genomes of AZS and DV901 to represent the S and L morphotype, respectively. Genes in the vicinity of the inversion breakpoints are shown and regions of the inversion are indicated with gray bars; inversion breakpoints are designated as A, B, C, and D to match the in-text description. Genes located at the inversion breakpoints are shaded darker than the other genes. Arrows indicate the locations of PCR primers listed in Table 1. Orthologs between the two morphotypes are designated with the same lower case alphabet (a – o). At the inversion breakpoints in the L morphotype, gene c’+l’ represents a PKS gene that is a fused product of the two PKS genes (genes c and l) that are at the margins of the inversion in the S morphotype genomes, and gene p is a cutinase gene that is not encoded in the S morphotype.

Synteny analysis revealed an inversion on chromosome 8 between the S and L morphotype genomes that is ≈531 kB and ≈551 kB in the L morphotype and S morphotype genomes respectively (Figures 2 and S1). Using AZS and DV901 to represent the S and L morphotype genomes respectively, the inversion contained 213 genes in AZS and 208 genes in DV901. Most genes in the inversion had orthologs between AZS and DV901, except for three genes that were identified as S morphotype-unique proteins (morphotype-unique protein results are presented below); one that was identified as an L morphotype-unique protein, and one that was present in S isolates in a region deleted in the L morphotype genomes but that was not detected in the morphotype-unique protein analysis. In the S morphotype genomes, the borders of the inversion affect two secondary metabolite gene clusters, one on each end. The inversion starts at the end of a polyketide synthase (PKS) gene (gene c and inversion breakpoint A in Figure 2) and ends at the end of another PKS gene (gene l and inversion breakpoint B in Figure 2). In the L morphotype genomes, the inversion starts at a PKS gene that is a fused product of the two PKS genes that are at the margins of the inversion in the S morphotype genomes (gene c’ + l’ and inversion breakpoint C in Figure 2), and ends within a cutinase gene that is not encoded in the S morphotype genomes (gene p and inversion breakpoint D in Figure 2). An exception to this occurs in the DV901 genome, where the cutinase gene was not predicted due to a SNP that resulted in a premature stop codon. The presence of the inversion was confirmed by PCR using the primers depicted at each inversion breakpoint in Figure 2 and listed in Table 1. Primer pairs used to detect inversion breakpoints A and B (Figure 2) only yielded amplicons in the S morphotype isolates and primer pairs used to detect inversion breakpoints C and D only yielded amplicons in the L morphotype isolates (data not shown).

In addition to synteny analysis, DELLY (Rausch *et al.*, 2012) revealed 31 deletions that are common to the S morphotype genomes relative to the L morphotype ranging in size from 307 bp – 12,939 bp, three of which were larger than 5kb. Manual inspection of deletions larger than 5kb revealed that the largest deletion was also present in L isolate MC04, and thus was removed from consideration. For the L morphotype genomes, there were 50 deletions relative to the S morphotype genomes that ranged in size from 151 bp – 14,979 bp, seven of which were larger than 5 kb. Manual inspection confirmed that all deletions larger than 5 kb were only present in the three L morphotype genomes.

L morphotype genes that were at the margins of, or within deletions larger than 1 kb in S morphotypes are listed in Table S4 (genes present in the L morphotype but missing in the S morphotype) using genes in DV901 to represent the L morphotype. Only one deletion in the S morphotype genomes overlapped with secondary metabolite clusters predicted by SMURF (Khaldi *et al.*, 2010) and this deletion is unlikely to impact differential gene expression, because the gene is non-functional in both S and L isolates. The *cypA* gene of the aflatoxin gene cluster, that encodes a cytochrome P450 monooxygenase required for aflatoxin G production (Ehrlich *et al.*, 2004) has deletions in both morphotypes and the deletion is larger by 575 bp in the S morphotype. L morphotype genes that were deleted in the S morphotype genomes also included genes that are predicted to encode proteins involved in carbon metabolism (a member of the glycosyl hydrolase family 12 and an alcohol dehydrogenase), nitrogen metabolism (a glutamine synthetase and a member of the NmrA-like protein family), and amino acid transport (a member of the proton-dependent oligopeptide transporter (POT) family) (Table S4).

S morphotype genes that were at the margins of, or within deletions larger than 1 kb in L morphotypes are listed in Table S5 (genes present in the S morphotype but missing in the L morphotype) using genes in AZS to represent the S morphotype. Seven deletions in the L morphotype genomes occurred within secondary metabolite gene clusters predicted by SMURF (Khaldi *et al.*, 2010). S morphotype genes that were deleted in the L morphotype included genes that encode proteins predicted to be involved in secondary metabolism (two polyketide synthases) and detoxification or biosynthesis of compounds (four cytochrome P450s). A schematic diagram of the two largest deletions in the S and L morphotype genomes are depicted in Fig. S2.

In addition to the deletions, DELLY (Rausch *et al.*, 2012) analysis revealed four inversions between the S and L morphotype genomes and one duplication in the L morphotype genomes; however manual inspection of these structural variants could not confirm their presence. There were no translocations that were detected by DELLY analysis (Rausch *et al.*, 2012).

### GO term enrichment and gene family expansion/contraction analyses

GO terms that were enriched in the genomes of each morphotype are listed in Table 3. S morphotype genomes were enriched in functions involved in secondary metabolism, such as isoquinoline alkaloid biosynthetic process (GO:0033075), (S)-stylopine synthase activity (GO:0047052), and N-methylcoclaurine 3′-monooxygenase activity (GO:0050593) that are involved in alkaloid biosynthesis or metabolism, as well as, branched-chain amino acid biosynthetic process (GO:0009082) and dihydroxy-acid dehydratase activity (GO:0004160) that can play a role in glucosinolate biosynthesis or in other pathways. S morphotype genomes were also enriched in processes generally involved in lipid metabolism including fatty acyl-CoA biosynthetic process (GO:0046949) and medium-chain fatty acid-CoA ligase activity (GO:0031956). In L morphotype genomes, enriched GO categories were exclusively involved in amino acid transport.

**Table 3:** GO terms enriched in S and L morphotype genomes

	GO ID	Name	Ontology source	p-value
S morphotype	GO:0009082	branched-chain amino acid biosynthetic process	biological_process	0.003
	GO:0004160	dihydroxy-acid dehydratase activity	molecular_function	0.003
	GO:0033075	isoquinoline alkaloid biosynthetic process	biological_process	0.021
	GO:0004737	pyruvate decarboxylase activity	molecular_function	0.021
	GO:0047052	(S)-stylopine synthase activity	molecular_function	0.021
	GO:0080092	regulation of pollen tube growth	biological_process	0.048
	GO:0004459	L-lactate dehydrogenase activity	molecular_function	0.048
	GO:0060148	positive regulation of posttranscriptional gene silencing	biological_process	0.048
	GO:0031956	medium-chain fatty acid-CoA ligase activity	molecular_function	0.048
	GO:0050593	N-methylcoclaurine 3′-monooxygenase activity	molecular_function	0.048
	GO:0046949	fatty-acyl-CoA biosynthetic process	biological_process	0.048
**L morphotype**	GO:0005313	L-glutamate transmembrane transporter activity	molecular_function	0.003
	GO:0015185	gamma-aminobutyric acid transmembrane transporter activity	molecular_function	0.003
	GO:0015813	L-glutamate transport	biological_process	0.003
	GO:0015812	gamma-aminobutyric acid transport	biological_process	0.003
	GO:0015180	L-alanine transmembrane transporter activity	molecular_function	0.003
	GO:0015808	L-alanine transport	biological_process	0.003
	GO:0015809	arginine transport	biological_process	0.004
	GO:0015189	L-lysine transmembrane transporter activity	molecular_function	0.006
	GO:0015819	lysine transport	biological_process	0.006
	GO:0015181	arginine transmembrane transporter activity	molecular_function	0.006

Comparison of protein families between S and L morphotype genomes revealed 68 protein families that are unique or expanded in the S morphotype genomes with six of these being unique to the S morphotype (Table 4). The hypergeometric test (p-value < 0.05) showed the DUF3589 protein family of unknown function (PF12141; p-value = 0.016) was significantly enriched in S morphotype genomes, with one protein containing two DUF3589 domains in the S morphotype, and none in the L morphotype genomes. CAZy annotation of the DUF3589 family protein predicts it is a β-1,2-mannosyltransferase and this is supported by sequence similarity to a β-mannosyltransferase of *Candida albicans* (KHC29884; 86% query coverage, 30% identity). S morphotype-unique protein families included proteins predicted to be involved in pyruvate metabolism (PF01326; pyruvate phosphate dikinase, PF00391 and PF028966; PEP-utilizing enzymes), an antifungal protein (PF11402), and a heme oxygenase (PF01126). S morphotype genomes also contained more copies of dehydratase (PF00920), chromate transporter (PF02417), ABC-2 transporter (PF01061), and ERG2 and Sigma 1 receptor like protein (PF04622) families.

**Table 4:** Protein families (Pfams) unique to or significantly expanded in S or L morphotype genomes. P-values (< 0.1) from the Pfam enrichment analysis are listed in the last column; p-values < 0.05 are in bold. The full list of protein families with expansions in each morphotype is listed in Table S2 and S3

	Pfam	Name	S morphotype			L morphotype			p-value
			AF12	AF70	AZS	BS01	DV901	MC04	
S	PF00391	PEP-utilizing enzyme, mobile domain	1	1	1	0	0	0	
morphotype	PF01326	Pyruvate phosphate dikinase, PEP/pyruvate binding domain	1	1	1	0	0	0	
	PF02896	PEP-utilizing enzyme, TIM barrel domain	1	1	1	0	0	0	
	PF01126	Heme oxygenase	1	1	1	0	0	0	
	PF11402	Antifungal protein	1	1	1	0	0	0	
	PF12141	Protein of unknown function (DUF3589)	2	2	2	0	0	0	**0.015**
	PF02417	Chromate transporter	6	4	4	2	2	2	0.06
L	PF00023	Ankyrin repeat	11	9	14	17	15	17	0.058
morphotype	PF00854	POT family	15	15	15	20	19	21	0.08
	PF01476	LysM domain	14	11	13	21	19	22	**0.009**
	PF01485	IBR domain, a half RING-finger domain	4	3	3	6	6	6	0.089
	PF12796	Ankyrin repeats (3 copies)	194	195	197	206	227	210	**0.041**

In L morphotype genomes, 59 protein families were expanded of which none were unique to the L morphotype (Table 4). The hypergeometric test showed the L morphotype genomes were significantly enriched in the LysM domain family (PF01476; p-value = 0.009) and Ankyrin repeats (3 copies) family (PF12796). LysM domains bind peptidoglycan or chitin, and in some plant pathogenic fungi LysM-containing proteins constitute effectors that allow evasion of host immune systems (Kombrink and Thomma 2013). Among protein families expanded in L morphotype genomes were the POT domain (PF00854) and amino acid permease (PF00324); the latter finding supports the GO analysis that showed enrichment in amino acid transporters. In addition, there were more copies of protein families predicted to be involved in nitrogen metabolism, and these include arginase (PF00491), serine aminopeptidase S33 (PF12146), peptidase family M28 (PF04389), prolyl oligopeptidase (PF00326), and trypsin (PF00089) protein families.

### Morphotype-unique proteins

**Figure 3:**
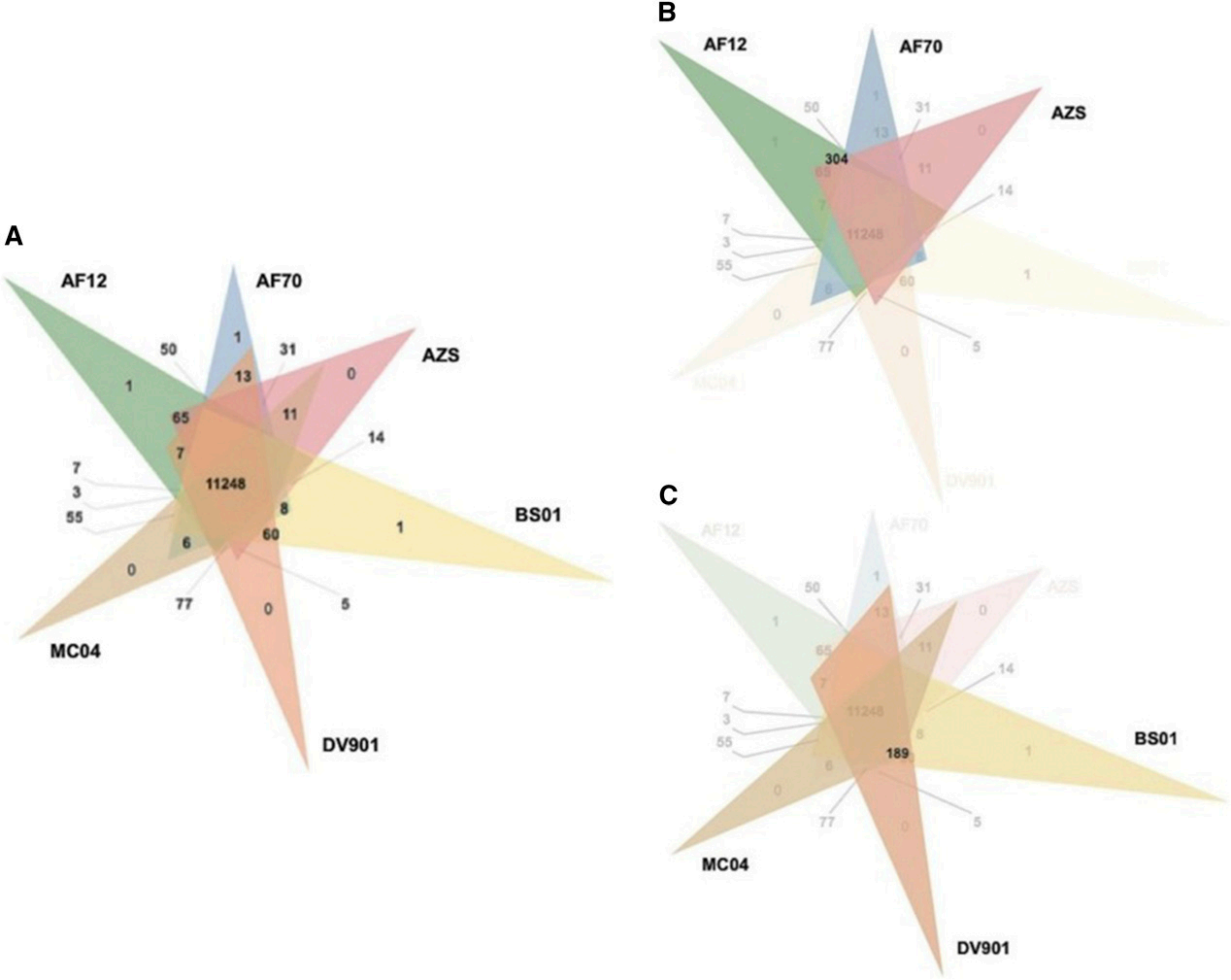
Venn diagrams representing the clustering results from Orthofinder to illustrate the number of proteins unique to and orthologous in each genome. (A) The number of clusters containing proteins unique to and orthologous in all six genomes; (B) the number of clusters containing proteins from only S morphotype genomes and (C) only L morphotype genomes.

**Figure 4:**
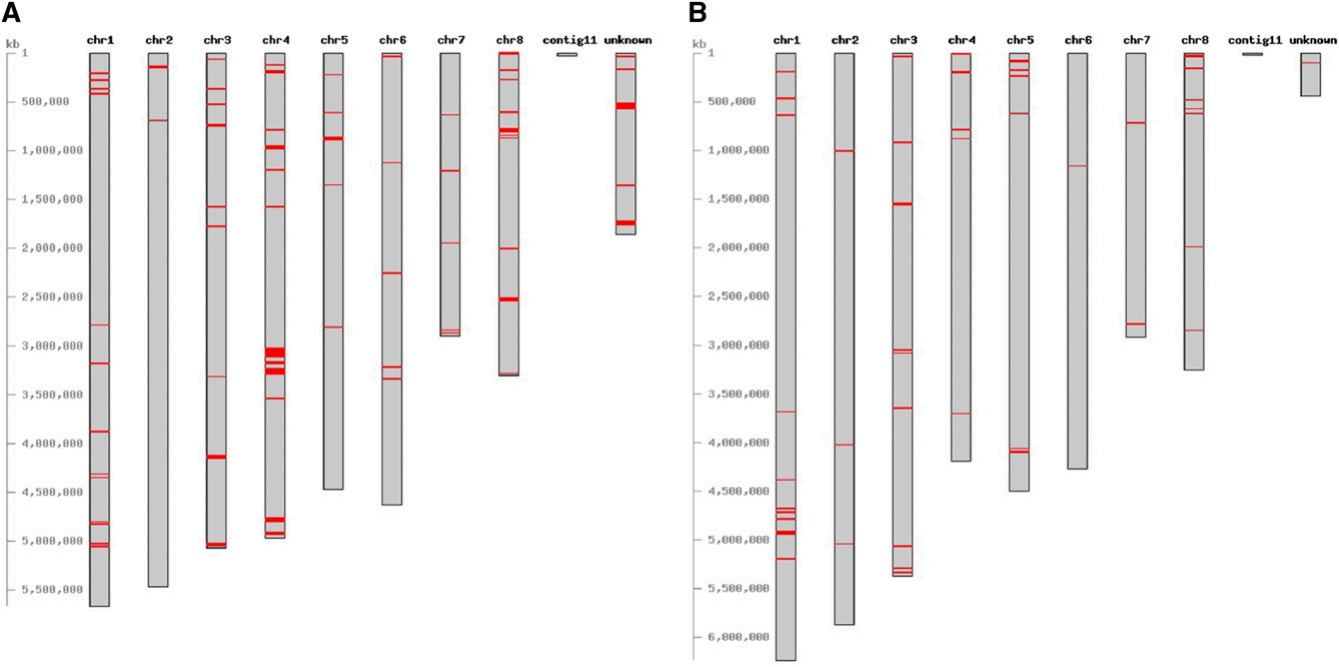
Chromosomal location of genes encoding morphotype-unique proteins (shown in red). Figures were created using CViT (Cannon and Cannon 2011). (A) Genes encoding S morphotype-unique proteins mapped on the pseudogenome of AZS. (B) Genes encoding L morphotype-unique proteins mapped on the pseudogenome of DV901.

To identify proteins unique to the S morphotype, clustering with Orthofinder (Emms and Kelly 2015) resulted in 304 clusters that consisted of proteins found only in the S morphotype genomes (Figure 3), and out of these, 279 were encoded by genes that were positionally conserved on contigs (positional orthologs). These clusters were filtered further by eliminating those that contained genes with significant BLASTn hits (≥80% identity and ≥80% query coverage) (Altschul *et al.*, 1990) against contigs of L morphotype genomes. This reduced the total to 191 proteins unique to the S morphotype genomes. Similarly, to identify proteins unique to the L morphotype, clustering with Orthofinder (Emms and Kelly 2015) resulted in 189 clusters that consisted of proteins from only L morphotype genomes (Figure 3), and out of these, 164 were encoded by positionally orthologous genes. Filtering the clusters further by BLASTn analysis (Altschul *et al.*, 1990) reduced the total to 82 proteins unique to the L morphotype genomes. Mapping the morphotype-unique proteins on the pseudogenomes of AZS and DV901 revealed they are distributed across all chromosomes in both morphotypes (Figure 4). Numbers of morphotype-unique proteins identified using this approach is likely an underestimate, because morphotype-unique proteins that are due to pseudogenization of genes in one morphotype from the accumulation of SNPs will not be detected due to retention of high sequence similarity between the genomes.

Morphotype-unique proteins with predicted Pfam, CAZy, MEROPS, and SMURF annotations are listed in Table S6 (S morphotype-unique proteins) and Table S7 (L morphotype-unique proteins). Below, we highlight morphotype-unique proteins that may play a role in niche adaptation, such as genes predicted to be involved in environmental response, gene regulation, secondary metabolism, and nutrient metabolism.

The S morphotype-unique proteins included proteins proposed to be involved in environmental responses; one is in the SUR7/PalI family (PF01349; a putative environmental sensor), and another has a CS domain (PF04969) predicted to bind to Hsp90, suggesting it is also a putative environmental response protein. S morphotype-unique proteins also included several involved in gene regulation; four are likely transcriptional regulators (containing the fungal specific transcription factor domain; PF04082), two may be involved in post-transcriptional regulation (belonging to the Shwachman-Bodian-Diamond syndrome protein family; PF01172, involved in RNA metabolism), and two may play a role in epigenetic regulation (an RNA dependent RNA polymerase; PF05183, and a protein with a SET domain; PF00856, involved in histone methylation). Other S morphotype-unique proteins were involved in nutrient metabolism. Proteins that play a role in carbon metabolism included an alcohol dehydrogenase (PF08240), an isocharismatase (PF00857), two glucose-methanol-choline (GMC) oxidoreductases (PF05199, PF00732), a lactate/malate dehydrogenase (PF02866, PF00056), and a pyruvate phosphate dikinase (PF01326). Proteins that play a role in nitrogen metabolism included an astacin (PF01400), a DJ-1/PfpI peptidase (PF01965), a fumarylacetoacetate (FAA) hydrolase (PF01557), and a carbon-nitrogen hydrolase (PF00795). In addition, the S morphotype had unique proteins that indicate a role in microbial competition. Those that may confer antimicrobial activity included a polyketide synthase (PF14765), an antifungal protein (PF11402), and a MAC/perforin (PF01823). Others that may play a role in detoxifying antimicrobials or toxic compounds produced by themselves or by others included six cytochrome P450s (PF00067), a glyoxylase/bleomycin resistance protein (PF00903), and a lipopolysaccharide kinase (PF06293).

The L morphotype-unique proteins contained a different set of regulatory proteins: transcriptional regulators including seven fungal specific transcription factors (PF04082, PF011951), putative post-transcriptional regulators (DEAD box RNA helicase; PF00270, PF00076), and epigenetic regulators (lysine methyltransferase; PF10294). Some L morphotype-unique proteins were involved in nutrient metabolism including carbon metabolism (alcohol dehydrogenase; PF08240), nitrogen metabolism (arginase; PF00491, trypsin; PF00089, and FAA hydrolase; PF01557), and lipid metabolism (acyl-CoA dehydrogenase; PF00441, PF02770, PF02771, and enoyl-CoA hydratase; PF00378). The L morphotype also had unique proteins that may confer antimicrobial activity (a protein with a Snoal-like domain; PF12680) or may detoxify compounds (RTA1-like protein; PF04479, cytochrome P450; PF00067).

### Genes under selection

Analysis by POTION (Hongo *et al.*, 2015) identified 12 genes under selection in S morphotype genomes and 2 genes under selection in L morphotype genomes (Table 5). Genes under selection in S morphotype genomes were predicted to encode two fungal specific transcription factors (PF11951, PF04082), a polyprenyl synthetase (PF00348), a HET protein (PF06985), a eukaryotic cytochrome b561 (PF03188), a eukaryotic elongation factor 5A hypusine (PF01287), and other proteins with unknown functions. These genes were distributed across chromosomes 1, 2, 3, 5, 6, 7, 8, and an unknown contig (assembled from contigs that did not map to *A. oryzae* RIB40). The eukaryotic elongation factor 5A hypusine gene under selection was located within the large inversion identified on chromosome 8. Genes under selection in L morphotype genomes were predicted to encode a sugar transporter (PF00083) and a protein with an unknown function. These genes were located on chromosomes 3 and 5.

**Table 5:** Genes under selection in S morphotype and L morphotype genomes and their q-values (corrected p-values for multiple testing) for the likelihood ratio tests using m12 and m78 nested site models

				Pfam	AF12/BS01 q-value		AF70/DV901 q-value		AZS/MC04 q-value	
	Genes under selection				m12	m78	m12	m78	m12	m78
S	AF12_05817	AF70_02866	AZS_06933	Fungal specific transcription factor domain	0.0056	0.0034	0.0047	0.0048	0.0066	0.0059
morphotype	AF12_08775	AF70_08873	AZS_09471	Fungal specific transcription factor domain	0.0000	0.0000	0.0000	0.0000	0.0000	0.0000
	AF12_10212	AF70_07962	AZS_10444	Polyprenyl synthetase	0.0040	0.0027	0.0049	0.0040	0.0372	0.0299
	AF12_08924	AF70_00133	AZS_07535	Heterokaryon incompatibility protein (HET)	0.0132	0.0130	0.0315	0.0245	0.0161	0.0152
	AF12_04453	AF70_04222	AZS_05654	Eukaryotic cytochrome b561	0.0040	0.0034	0.0047	0.0049	0.0042	0.0035
	AF12_04574	AF70_04341	AZS_05775	No annotation	0.0000	0.0000	0.0000	0.0000	0.0000	0.0000
	AF12_11547	AF70_11889	AZS_12064	Eukaryotic elongation factor 5A hypusine, DNA-binding OB fold	0.0040	0.0034	0.0047	0.0049	0.0042	0.0043
	AF12_12244	AF70_01569	AZS_12604	No annotation	0.0005	0.0005	0.0004	0.0003	0.0003	0.0003
	AF12_12631	AF70_06345	AZS_08864	No annotation	0.0000	0.0000	0.0000	0.0000	0.0000	0.0000
	AF12_11962	AF70_12568	AZS_12704	No annotation	0.0343	0.0252	0.0047	0.0035	0.0039	0.0025
	AF12_02667	AF70_03436	AZS_10736	Protein kinase domain	0.0030	0.0023	0.0039	0.0040	0.0032	0.0027
	AF12_03527	AF70_04870	AZS_05363	No annotation	0.0010	0.0008	0.0011	0.0011	0.0010	0.0003
L	BS01_06841	DV901_08350	MC04_12622	Sugar (and other) transporter	0.0129	0.928	0.0077	0.8656	0.0108	0.0082
morphotype	BS01_09387	DV901_12827	MC04_12342	No annotation	0.0004	0.0003	0.0005	0.0005	0.0006	0.001

### Differences in secondary metabolite clusters, antibiotic resistance proteins, CAZy, and MEROPS peptidases between morphotypes

**Figure 5:**
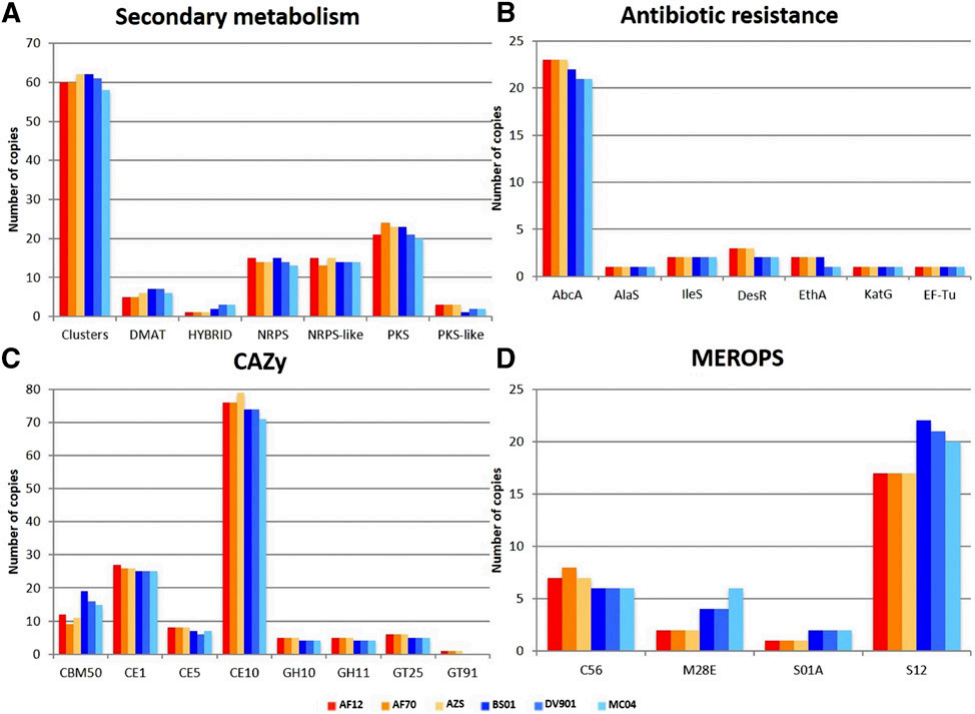
Differences in copy numbers of proteins involved in secondary metabolism, antibiotic resistance, carbohydrate metabolism, and peptidase activity between S and L morphotype genomes. (A) Number of secondary metabolite clusters and their backbone genes predicted in each genome by SMURF (Khaldi *et al.*, 2010). Abbreviations: prenyltransferases (DMAT), nonribosomal peptide synthase (NRPS), polyketide synthase (PKS), hybrid NRPS-PKS enzymes (HYBRID). (B) Number of proteins predicted to confer antibiotic resistance in each genome by CARD (Jia *et al.*, 2017). Abbreviations: multidrug resistant ABC transporter (AbcA), aminocoumarin resistant (AlaS), Bifidobacteria intrinsic *ileS* conferring resistance to mupirocin (IleS), β-Glycosidase involved in *Streptomyces venezuelae* self-resistance (DesR), *Mycobacterium tuberculosis ethA* with mutation conferring resistance to ethionamide (EthA), *M. tuberculosis katG* mutations conferring resistance to isoniazid KatG, *S. cinnamoneus* elongation factor thermo unstable mutants conferring resistance to elfamycin (EF-Tu). (C) CAZy categories (Yin *et al.*, 2012) with differences in copy numbers between S and L morphotype genomes. Abbreviations: carbohydrate-binding modules (CBM), carbohydrate esterases (CE), glycoside hydrolases (GH), glycosyltransferases (GT). (D) MEROPS categories (Rawlings *et al.*, 2012) with differences in copy numbers between S and L morphotype genomes. Abbreviations: PfpI endopeptidase family/cysteine peptidase (C56), aminopeptidase Y family/metallo peptidase (M28), chymostrypsin family/serine peptidase (S01), D-Ala-D-Ala carboxypeptidase B family/serine peptidase (S12).

The number of secondary metabolite clusters and their backbone genes that were predicted by SMURF (Khaldi *et al.*, 2010) are shown in Figure 5A. There was no notable difference in the number of secondary metabolite clusters between morphotypes, but S morphotype genomes contained 1 – 2 more copies of PKS-like proteins and L morphotype genomes contained 1 – 2 more copies of HYBRID PKS-NRPS proteins. Ten S morphotype-unique proteins belonged to five secondary metabolite clusters and three L morphotype-unique proteins belonged to three secondary metabolite clusters, indicating compositions of several secondary metabolite clusters differ between morphotypes.

The number of proteins predicted to confer antibiotic resistance in S and L morphotype genomes is shown in Figure 5B. Comparing annotations to the comprehensive antibiotic resistance database (CARD) revealed that S morphotype genomes have an additional copy of DesR; a protein involved in self-resistance to antibiotics produced in *Streptomyces venezuelae*, and 1 – 2 additional copies of AbcA; an ABC transporter that can confer resistance to methicillin, daptomycin, cefotaxime, and moenomycin (Jia *et al.*, 2017). The remaining CARD categories had the same number of copies in the genomes of both morphotypes. None of the proteins annotated by CARD belonged to the morphotype-unique protein set, but this is likely because a conservative approach was used to identify morphotype-unique proteins.

Carbohydrate active enzyme (CAZy) categories that were present in different copy numbers in S and L morphotype genomes are represented in Figure 5C. Comparison of CAZy annotations revealed that S morphotype genomes contain a unique β-1,2-mannosyltransferase (GT91) that was also identified in the morphotype-unique protein analysis. In addition, the S morphotype genomes contained more copies of three carbohydrate esterase families: CE1, CE5 that contain cutinases, and CE10 that acts on non-carbohydrate substrates. The S morphotype also contains more members of three glycoside hydrolase families: GH10 and GH11 that contain xylanases, and GH25 that contain lysozymes. L morphotype genomes did not contain any morphotype-unique CAZy categories, but contained more copies of carbohydrate-binding module family 50 domains that bind to peptidoglycan and chitin (CBM50).

Peptidase and peptidase inhibitor (MEROPS) categories that were present in different copy numbers in S and L morphotype genomes are represented in Figure 5D. Comparison of MEROPS annotations revealed that the S morphotype genomes contained more copies of PfPI endopeptidase (C56) family, whereas the L morphotype genomes contained more copies of aminopeptidase Y (M28E), chymotrypsin (S01A), and D-Ala-D-Ala carboxypeptidase B (S12) families.

### Phylogenetic analysis

**Figure 6:**
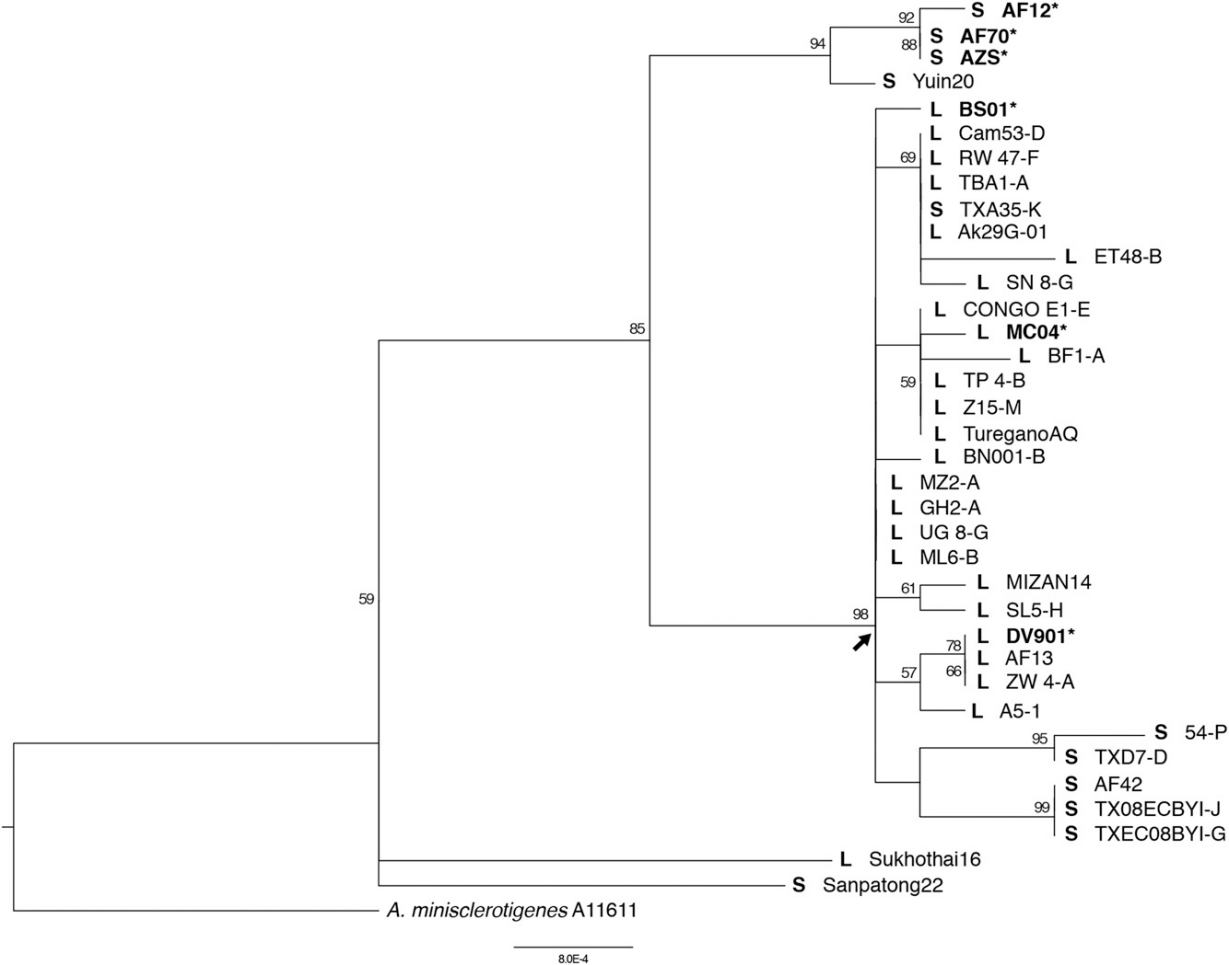
Maximum likelihood (ML) tree based on concatenated sequences of the 5′ half of *tsr1*, 3′ half of *tsr1*, and *cmd* (3.4 kb total). ML bootstrap values (>50%) are indicated at the nodes and the arrow indicates the major clade referred to in-text. *Aspergillus minisclerotingenes* was used as the outgroup; isolates used in this analysis are listed in Table 1. Isolates of *A. flavus* are indicated with an S or L in bold to represent S or L morphotype, respectively, and isolates used in the genomics analyses are both in bold and marked with an asterisk.

A maximum likelihood tree with clade support values (>50%) is presented in Figure 6. The S isolates used in the current genomic analyses along with S isolate Yuin20 formed a distinct clade from most other *A. flavus* isolates with high support. The major clade (indicated with the arrow in Figure 6) included all L isolates except Sukhothai16, as well as some S isolates; five of these S isolates formed a subclade. The three L isolates used for the genomic analyses are present in this major clade, distinct from the S isolates used in the genomic analyses. The number of polymorphic sites was low among all the *A. flavus* isolates and thus resolution within the major clade was limited. Two isolates, L isolate Sukhothai16 and S isolate Sanpotong22, placed separate from these described clades.

## Discussion

Although *A. flavus* is generally considered a soil-borne fungus, the differences in asexual reproductive strategy and levels of aflatoxin production between S and L morphotypes suggest that the S morphotype, producing large numbers of sclerotia and consistently toxigenic is adapted to survival in the soil, and the L morphotype, with large numbers of conidia and variable levels of aflatoxin is adapted to aerial dispersal to the phyllosphere (Cotty *et al.*, 1994). The closest sister taxa to *A. flavus* are *A. minisclerotigenes* and *A. parvisclerotigenus* (Pildain *et al.* 2008), both of which have a morphology similar to the S morphotype. Therefore, we believe the morphology of the S morphotype is ancestral to *A. flavus*, and it is speculated that the two morphotypes diverged 1-3 million years ago (Ehrlich *et al.* 2005). To test the hypothesis that S and L morphotypes are differentially adapted to the soil and phyllosphere respectively, we sequenced the genomes of three S isolates and three L isolates and identified both structural and gene content differences that we propose play a role in differential niche adaptation.

These analyses identified 191 proteins unique to the S morphotype and 82 proteins unique to the L morphotype. This is likely an undercount as our approach was conservative to avoid false positives due to misannotation. Morphotype-unique genes were present on each of the eight chromosomes, indicating acquisition was not through a single recombination or transfer event. Morphotype-unique proteins included those involved in environmental response, transcriptional regulation, post-transcriptional regulation, epigenetic regulation and metabolic potential. These differences would allow the two morphotypes to selectively respond to conditions critical to the soil and phyllosphere, which differ in nutrient availability, competition within the niches, and their ambient conditions.

Morphotype-unique proteins in the S morphotype are involved in microbial competition, resilience against toxic compounds, and nutrient acquisition; these traits are advantageous for survival in the highly competitive soil environment. One of the protein families that the S morphotype gained is an antifungal protein with high amino acid similarity to the well-studied protein Pc24g00380/PAF in *Penicillium chrysogenum* (98% query coverage, 68% identity, e-value 4e-38; Marx *et al.*, 1995) that has antifungal activity against diverse fungal taxa (Kaiserer *et al.*, 2003; Galgóczy *et al.*, 2005; Galgóczy *et al.*, 2007; Barna *et al.*, 2008; Galgóczy *et al.*, 2008). Unlike the microbial diversity present in soils, the phyllosphere community consists predominantly of bacteria, with the diversity and numbers of fungi expected to be lower (Vorholt 2012); therefore the antifungal protein in the S morphotype would be more advantageous for soil fungi. In support of this, yeasts isolated from the soil have greater antagonistic activity against filamentous fungi than yeasts isolates from the phyllosphere (Hilber-Bodmer *et al.*, 2017).

Heavy metal tolerance is also important to the adaptive evolution of soil dwelling organisms (Ryan *et al.*, 2009; Chan *et al.*, 2016; Faddeeva-Vakhrusheva *et al.*, 2016). S isolates have two or more additional copies of the chromate transporter family (PF02417) than L isolates, which could confer increased tolerance to chromate. In addition, the S morphotype may have gained increased resistance to antifungals by having two copies of the C-8 sterol isomerase (PF04622; ERG2 and Sigma 1 receptor-like family) in contrast to the single copy in the L morphotype. Ergosterol biosynthesis enzymes, C-8 sterol isomerase and C-14 sterol reductase, are targets for amine antifungals produced by many microbes including soil bacteria (Trejo and Bennett 1963; Vicente *et al.*, 2003; Hull *et al.*, 2012; Vincent *et al.*, 2013; Sanglard 2016). In *Fusarium graminearum*, possession of a second copy of C-14 sterol reductase confers increased resistance to amine fungicides (Liu *et al.*, 2011). Similarly, the additional copy of C-8 sterol isomerase in the *A. flavus* S morphotype may enhance resistance to antifungal compounds produced by soil microbes enabling it to compete in the soil niche.

In the soil, nutrient availability is influenced by root exudates that contain organic acids, sugars and amino acids, as well as by degradation of organic matter by microorganisms (Rengel and Marschner 2005; Turner *et al.*, 2013; Dotaniya and Meena 2015). Among the protein families that were gained in the S morphotype were a pyruvate phosphate dikinase and a lactate dehydrogenase encoded by adjacent genes, which could allow the fungus to take advantage of diverse carbon sources in the soil. Lactate dehydrogenase catalyzes the conversion of L-lactate to pyruvate, and pyruvate phosphate dikinase phosphorylates pyruvate to produce phosphoenolpyruvate. These two enzymes could allow the S morphotype to perform gluconeogenesis from lactate to utilize organic acids in the soil as an energy source. Although the soil may have more available carbon sources compared to the phyllsophere, iron is a scarce nutrient due to lack of mobility (Rengel and Marschner 2005). Therefore, iron acquisition strategies independent of siderophore scavenging are adaptive traits that support adequate iron uptake in soil (Mathew *et al.*, 2014; Zhu *et al.*, 2016). Although both morphotypes have siderophore genes (data not shown), only the S morphotype has a heme oxygenase, which catalyzes the release of iron from heme providing increased capacity to competitively acquire iron.

In contrast to the S morphotype, we hypothesize the L morphotype is adapted to the phyllosphere, which is protected by a cuticle layer that limits diffusion of compounds and thus is scarce in nutrients (Schönherr and Baur 1996; Lindow and Brandl 2003; Bringel and Couée 2015). In epiphytic bacteria, the ability to scavenge for limited substrates has been implicated in adaptation to the phyllosphere and their metagenomes contain a prominence of transporter genes (Delmotte *et al.*, 2009). Similarly, L morphotype genomes have a significant GO term enrichment of amino acid transporters as well as an expansion of POT (proton-dependent oligopeptide transporter) family proteins. In addition to transporters, the L morphotype possesses a second copy of arginase, which catalyzes the breakdown of arginine into ornithine and urea. Arginine constitutes a major storage and transport form of organic nitrogen in plants (Winter *et al.*, 2015) and is shown to decrease significantly on the phylloplane in the presence of certain epiphytic bacteria (Ryffel *et al.*, 2016). Therefore, the presence of an additional copy of arginase in the L morphotype may aid in competitive nitrogen acquisition in the presence of these bacteria, allowing the fungus to take advantage of the proteinogenic amino acid with the highest nitrogen to carbon ratio (Winter *et al.*, 2015). The L morphotype’s ability to scavenge for nitrogen sources was also reflected in the expansion of three MEROPS peptidase categories.

In addition to the prominence of unique proteins involved in nitrogen metabolism, proteins from the L morphotype were significantly enriched in the LysM domain (PF01476), which binds peptidoglycan or chitin. Proteins containing LysM domains can be categorized as either proteins that contain one or more LysM domains plus a chitinase domain or as proteins with multiple LysM domains and no chitinase domain. The first type is involved in fungal growth by creating plasticity in cell walls and the second type serves to evade triggering microbe-associated molecular pattern (MAMP) elicited immune responses by binding chitin particles released from hyphae (de Jonge and Thomma 2009; Kombrink and Thomma 2013; Martinez *et al.* 2012). L morphotype-unique LysM proteins contain two to three LysM domains, but lack a chitinase domain indicating they belong to the latter type. These proteins could allow the L morphotype to colonize the phyllosphere while evading the host immune system.

Chromosomal rearrangements have been proposed to play major roles in evolution that lead to differential adaptation (Schmidt and Hensel 2004; Kirkpatrick 2010; Kirkpatrick and Barton 2006; Stukenbrock 2013; Guttman *et al.*, 2014; Leducq 2014; Raeside *et al.*, 2014; Seidl and Thomma 2014); A notable structural difference between the S and L morphotype genomes is a >530 kB inversion on chromosome 8. Inversions are major drivers of adaptation and speciation in various organisms (Kirkpatrick and Barton 2006; Hoffmann and Rieseberg 2008; Kirkpatrick 2010) by impacting homologous chromosome pairing during meiosis that can result in reduced fertility and reproductive isolation (Hoffmann and Rieseberg 2008). Our phylogenetic analysis of *A. flavus* S and L morphotype taxa from diverse geographic regions does not resolve whether the morphotypes form monophyletic clades due to limited sample size, but does indicate the S and L isolates used in the genomic analyses belong to two distinct clades. The inversion may have resulted in limited gene flow between the morphotypes leading to distinct lineages. Inversions can also result in fortuitous changes via gene disruption or altered gene regulation at the breakpoints (Kirkpatrick and Barton 2006). In the S morphotype, there are two secondary metabolite gene clusters at the inversion breakpoints; if the compounds produced by these clusters are advantageous for microbial competition in the soil, the S inversion configuration would be advantageous for soil survival. In contrast, in the L morphotype, one of the inversion breakpoints encodes a cutinase gene, which may be favorable for nutrient acquisition in the phyllosphere. Finally, inversions can suppress recombination within the inverted region leading to maintenance of divergent allele combinations that could be advantageous in contrasting niches (Kirkpatrick and Barton 2006; Hoffmann and Rieseberg 2008; Kirkpatrick 2010). In both the S and L morphotypes, the inverted region contains genes under selection and morphotype-unique genes. In the inverted region of the S morphotype, a eukaryotic elongation factor 5A hypusine gene is under selection-and four S morphotype-unique proteins are present (a cytochrome p450, a hexapeptide repeat of succinyl transferase, a UbiA prenyltransferase, and an unknown protein). The L morphotype has one unique protein (protein with an FAD binding domain) in the inverted region. The region may also contain genes with adaptive allelic differences between the morphotypes that were not identified to be under selection. Suppressed recombination within the inversion could maintain these gene and allelic differences between the morphotypes. However, elucidation of mechanisms and extent to which these differences play a functional role in niche adaptation will require validation.

In addition to the inversion creating differences in secondary metabolite clusters, the number of deletions within secondary metabolite clusters also differs between the morphotypes with more intact clusters present in the S morphotype. The L morphotype contains seven deletions within secondary metabolite clusters relative to the S morphotype, including the deletion of two PKS genes. In contrast, the S morphotype contains one deletion within secondary metabolite clusters relative to the L morphotype; however, this deletion is within the *cypA* gene of the aflatoxin gene cluster in which both S and L isolates have deletions with the S isolates having a larger deletion. Therefore, both morphotypes have defective *cypA* genes resulting in the loss of aflatoxin G production (Ehrlich *et al.*, 2004; Probst *et al.*, 2012; Probst *et al.*, 2014; Adhikari *et al.*, 2016). The differences in deletions within secondary metabolite clusters suggest the S morphotype is under higher selective pressure to maintain secondary metabolite production, consistent with the pressure to maintain aflatoxin production. Polyketide biosynthetic genes are enriched in metagenomes of soil bacteria as well (Tringe *et al.* 2005). The extra intact clusters in the S morphotype may produce antimicrobial compounds, signaling molecules, or chelating agents (Demain and Fang 2000) that could be advantageous for competition against the more plentiful and diverse microbes in the soil compared to the phyllosphere (Delmotte *et al.*, 2009; Knief *et al.*, 2012).

In summary, we have used comparative genomics to test the hypothesis that the S and L morphotypes have genetic differences that could play a role in differential adaptation to the soil and phyllosphere, respectively. Our genomic comparisons indicate there are differences in gene regulation, antimicrobial activity, resistance to natural compounds and toxic chemicals, carbon and nitrogen metabolism, iron acquisition, and secondary metabolite production between the morphotypes. We have proposed how these differences could be advantageous for niche adaptation and have provided a foundation for experiments to determine the relevance of the genomic differences in niche adaptation. Functional validation of these genes is necessary as well as examination of additional S and L isolates to extend these results. Furthermore, many of the morphotype-unique proteins did not have predicted functions or domains, therefore, functional analysis of these via knockout or knock-in experiments would help in defining their potential roles in niche adaptation of the two morphotypes.
